# Overexpression of U three protein 14a (UTP14a) is associated with poor prognosis of esophageal squamous cell carcinoma

**DOI:** 10.1111/1759-7714.13176

**Published:** 2019-09-08

**Authors:** Kun‐Kun Li, Cheng‐Yi Mao, Jing‐Ge Zhang, Qiang Ma, Ying‐Jian Wang, Xue‐Hai Liu, Tao Bao, Wei Guo

**Affiliations:** ^1^ Department of Thoracic Surgery Daping Hospital, Army Medical University Chongqing China; ^2^ Department of Pathology Daping Hospital, Army Medical University Chongqing China

**Keywords:** Esophageal squamous cell carcinoma, survival, U three protein 14a

## Abstract

**Background:**

Esophageal squamous cell carcinoma (ESCC) is one of the most aggressive and lethal cancers lacking valid prognostic biomarkers. As an essential component of a large ribonucleoprotein complex, U Three Protein 14a (UTP14a) might play important roles in human tumorigenesis. However, the clinical significance and functions of UTP14a in ESCC still remain unclear.

**Methods:**

From September 2009 to August 2015, 210 patients with ESCC of the thoracic esophagus underwent thoracoscopic esophagectomy in our institute. The corresponding 210 tissue samples and 30 cancer‐distant mucosa (CDM) samples were tested for UTP14a expression by immunohistochemical staining. The long‐term survival was analyzed by the Kaplan–Meier method and Cox proportional hazards regression analyses. CCK8, cell colony formation, cell cycle, apoptosis, cell invasion, and wound healing assays were carried out with ECA109 cells to evaluate the effects of UTP14a on ESCC in vitro.

**Results:**

UTP14a was positively expressed in 88.1% (185/210) of the ESCC samples. UTP14a expression in ESCC was significantly higher than in CDM, as further confirmed by Western blot analysis. High expression of UTP14a in ESCC correlated significantly with tumor invasive depth (pT stage), which predicts poor disease‐free survival and disease‐specific survival, as indicated by the log‐rank test and Cox proportional hazards regression analysis. Additionally, our in vitro experiments further demonstrated that knockdown of UTP14a inhibits cell proliferation and invasion in ECA109 cells.

**Conclusions:**

Our results suggest that UTP14a is aberrantly expressed in ESCC, plays a critical role in cancer progression and could be a potential prognosis predictor of ESCC.

## Key points

### Significant findings of the study

High expression of UTP14a in ESCC correlated significantly with tumor invasive depth and predicts poor survival; in vitro experiments demonstrated that UTP14a inhibits cell proliferation and invasion.

### What this study adds

The results suggest that UTP14a could be a potential prognosis predictor of ESCC.

## Introduction

Esophageal cancer (EC) is one of the most aggressive and lethal cancers in patients, and it ranks seventh in terms of incidence and sixth in mortality globally.^1^ The histological type of esophageal cancer most often found in Western countries is adenocarcinoma, whereas in China, EC mainly occurs in the form of esophageal squamous cell carcinoma (ESCC).^2^ Although recent improvements in surgical technology and multidisciplinary treatments could result in better outcomes, the five‐year overall survival rate of ESCC remains low (20.9%–43.5%), because of the high incidence of local recurrence and metastasis^3^, ^4^; recurrence and metastasis can even occur in the early stages. Numerous molecular and protein abnormalities have been found in the pathogenesis of ESCC. However, disease‐specific and effective biomarkers have not yet been found, and sensitive biomarkers, as well as novel therapeutic targets, remain to be identified.

In our previous research, we found that wild‐type p53‐induced gene 1 (WIG‐1) could effectively reverse the malignant phenotype, suppress cell proliferation, and induce G0/G1 cell cycle arrest and apoptosis in EC109 cells under zinc deficiency conditions.^5^, ^6^ Furthermore, by yeast‐two‐hybrid analysis we showed that U three protein 14a (UTP14a) directly binds to WIG‐1; UTP14a might therefore be a direct downstream target of WIG‐1 (K.K. Li *et al*. unpublished data).

UTP14a is an essential component of a large ribonucleoprotein complex bound to the U3 small nucleolar RNA. It plays a key role in ribosome biogenesis and 18S rRNA synthesis.^7^, ^8^ Hu *et al*.^9^ reported that UTP14a is overexpressed in various kinds of tumor cell lines, leading to cell cycle arrest and apoptosis through p53 protein degradation, and moreover, ectopically expressed UTP14a could protect tumor cells from chemotherapeutic drug‐induced apoptosis.^10^ However, the transcription of UTP14a is not regulated by p53.^11^ The expression of UTP14a is upregulated in human hepatocellular carcinoma and colorectal carcinoma, and may be associated with poor prognosis.^12^, ^13^ These previous studies suggest that UTP14a plays important roles in human tumorigenesis.

In this study, we investigated the expression of UTP14a in ESCC and cancer‐distant mucosa (CDM), evaluated the relationship between UTP14a expression and clinicopathological characteristics and survival time of patients with ESCC, and examined whether UTP14a is a potential prognostic biomarker for ESCC.

## Methods

### Patients, specimens and cell lines

From September 2009 to August 2015, 210 patients with ESCC of the thoracic esophagus underwent thoracoscopic esophagectomy (McKeown) in the Department of Thoracic Surgery, Daping Hospital, Army Medical University (Chongqing, China). All surgeries were performed by the same surgeon (W.G.). The postoperative TNM stage was classified in accordance with the eighth edition of the American Joint Committee on Cancer (AJCC) staging protocol.^14^ The corresponding 210 formalin‐fixed and paraffin‐embedded tissue samples were obtained from the Department of Pathology. During surgery, routine intraoperative frozen section examination was performed to obtain a negative surgical margin (NE). All NE samples were verified to be tumor‐free by hematoxylin and eosin (H&E) staining by a pathologist after surgery; 30 of these matching NE samples were selected and served as a control. The study was approved by the Ethics Committee of Daping Hospital.

Exclusion criteria was as follows: (i) neoadjuvant therapy, (ii) postoperative death (death within 30 days after surgery or before discharge from the hospital), (iii) loss to follow‐up, (iv) noncurative (R1 or R2) resection (tumor‐free margin <1 mm), and (v) death due to causes unrelated to ESCC.

The esophageal cancer cell line ECA‐109 was obtained from GeneChem (Shanghai, China) in 2017. The cell line was tested and authenticated by short tandem repeat (STR) analysis as described in the ANSI Standard (ASN‐0002) by the American Type Culture Collection (ATCC) Standards Development Organization (SDO) in 2017.

### Tissue microarrays, immunohistochemistry and Western blot analysis

A tissue microarray containing a total of 210 formalin‐fixed and paraffin‐embedded tissue samples was constructed, and immunohistochemistry (IHC) was performed as previously described.^15^ Briefly, 4 μm thick sections were deparaffinized in xylene and gradually rehydrated in alcohol solutions. After normal antigen retrieval, IHC staining using the Histostain‐Plus Kit and DAB kit (DKAO/Agilent, Denmark) was performed following the manufacturer's instructions. Subsequently, rabbit anti‐UTP14a polyclonal antibody (1:200, Affinity, USA) was used at 4°C. Slides were counterstained with light hematoxylin, dehydrated, and cover‐slipped.

All slides were assessed by two pathologists (Qiang Ma and Chengyi Mao, Department of Pathology, Daping Hospital, Army Medical University) without any knowledge of the clinicopathological features. Disagreements were resolved by discussion. A semiquantitative scoring system in considering the staining intensity and proportion was used to estimate the expression of UTP14a as described previously.^15^ Each slide was evaluated by the intensity of the cytoplasmic staining (no staining = 0, weak staining = 1, moderate staining = 2, and strong staining = 3) and the proportion of stained cells (0% = 0, 1–30% = 1, 31–60% = 2, and 61–100% = 3), and the sum ranging from 0 (the minimum score) to six (the maximum score) was the final immunoreactive score, classified as negative expression (0), weak expression (1–2), moderate expression (3–4), and strong expression (5–6). We considered the final immunoreactive score was 1 or higher to be positive.

Proteins from freshly frozen tissue samples were extracted using the T‐PER Tissue Protein Extraction kit (Thermo Scientific, USA), separated on SDS‐PAGE (Beyotime, China), and transferred onto a polyvinylidene difluoride (PVDF) membrane (Millipore, USA) for Western blot analysis. The membrane was then incubated with rabbit anti‐UTP14a polyclonal antibody (Affinity, US). GAPDH (Cell Signaling Technology, USA) was used as an internal control. Protein bands were analyzed with Quantity One software.

### Quantitative real‐time PCR (qRT‐PCR) analysis

Total RNA was extracted from cells using Trizol reagent (Shanghai Pufei, China) and reverse‐transcribed into cDNA with the M‐MLV reagent kit (Promega, USA). qRT‐PCR analysis was conducted with SYBR Master Mixture (Takara, USA) to measure the expression of UTP14a. GAPDH was used as an internal control, and the relative mRNA expression of UTP14a mRNA was calculated using the 2^−ΔΔCt^ method.^16^

UTP14a primers: forward, 5'‐AATAAAACCGCACAAGTCC‐3′, and reverse, 5'‐ACAGGGGTCAGTAAAGGGT‐3′; product size: 218 bp.

GAPDH primers: forward, 5'‐TGACTTCAACAGCGACACCCA‐3′, and reverse, 5'‐CACCCTGTTGCTGTAGCCAAA‐3′; product size: 121 bp.

### Plasmids and transfection of cells

GV248 lentivirus was used to express small interfering RNAs (siRNAs) targeting the UTP14a sequence (Genbank no. NM_006649) (UTP14a‐siRNA lentivirus). A nontargeting sequence was purchased from GeneChem (Shanghai, China) and used as a negative control (NC). The template of the siRNA was 5'‐GAGCAGCTGCGGAAGGTTAAT‐3′. The sequences were cloned into the GV248 lentivirus (GeneChem, Shanghai, China) to generate the lentiviral vectors. ECA109 cells were infected with UTP14a‐siRNA lentivirus and NC lentivirus. The interference efficiency of the template was measured by qRT‐PCR.

### Cell colony formation assay and CCK8 assay

The proliferation of ECA109 cells infected with LV‐UTP14A‐RNAi lentivirus (KD) and NC lentivirus was assessed by cell colony formation and cell counting kit 8 (CCK8) assays.

In the cell formation assay, ECA109 cells suspended in DMEM (Corning, USA) containing 10% fetal bovine serum were plated in six‐well plates at 1000 cells/well. The plates were incubated for 14 days and colonies with more than 50 cells were counted. The cells were then stained by crystal violet dye (Sangon Bio, China) and counted with a microscope. Each sample was done in triplicate, and the experiments were repeated three times.

The CCK8 assay was carried out following the manufacturer's instructions (Sigma, USA). Absorbance of each well was quantified at 450 nm by an enzyme‐linked immunosorbent assay (ELISA) microplate reader, and cell growth was assayed every day for five days.

### PI‐FACS cell cycle analysis and apoptosis

Cells were infected with LV‐UTP14A‐RNAi lentivirus (KD) or NC lentivirus. The various cell cycle phases were determined using propidium iodide (Sigma, USA). Cell populations in the G0/G1 phase, S phase, and G2/M phase were measured using flow cytometry and plotted against cell volume. Data are reported as mean ± standard deviation of three independent experiments. Cell apoptosis was assayed by staining with Annexin V‐APC (eBioscience, USA), following the manufacturer's instructions, and counting the cells by flow cytometry.

### Cell invasion assay

Cell invasion was studied by using BioCoat Matrigel Invasion Chambers (Corning, USA). After 72 hours, cells migrating through the membrane were stained by Giemsa (Shanghai Dingguo Bio, China) and counted with a microscope. Nine random visual fields (×200 magnification) were chosen for each membrane, and the results expressed as number of migratory cells per field. Data are reported as the mean ± standard deviation of three independent experiments.

### Celigo wound healing assay

The Celigo cytometer (Nexcelom, USA) provided a tool to directly measure cell migration for label‐free or fluorescently labeled cells. We chose a 96‐well format, and the wound was created by a square mask. After 24 hours, the entire well was imaged, and the number of cells or percentage of confluence within the previously cleared region was measured. The migration rate is reported as migrated cell surface area (24 hours)/wound area (0 hours) × 100%. Data are reported as the mean ± standard deviation of five independent experiments.

### Follow‐up

All 210 patients were followed‐up in the outpatient clinic after surgery every three months during the first year and every six months until the fifth year; subsequent follow‐ups were performed annually.^4^, ^17^ The follow‐up information was completed until March 2018 or death. The follow‐up duration ranged between 3.8 and 99.6 months (median, 36 months). The disease‐free survival (DFS) rate was established using the survival from surgery to the date of recurrence. Disease‐specific survival (DSS) was established using the survival from surgery to the date of death due to ESCC.

### Statistical analysis

Numerical data are expressed as the mean ± standard deviation. Continuous and categorical variables were compared by using the Student *t*‐test and the χ^2^ test, quantitative variables were compared by one‐way analysis of variance (ANOVA). Patient survival curves were constructed by the Kaplan–Meier method. The log‐rank test was used to compare survival differences between groups for each variable. Univariate and multivariate Cox proportional hazards regression analyses were performed to identify potential prognostic factors. All statistical calculations were performed using SPSS statistical software, version 22.0 (IBM SPSS, USA). A *P*‐value of <0.05 was considered statistically significant.

## Results

### UTP14a expression in ESCC and CDM

**Figure 1:**
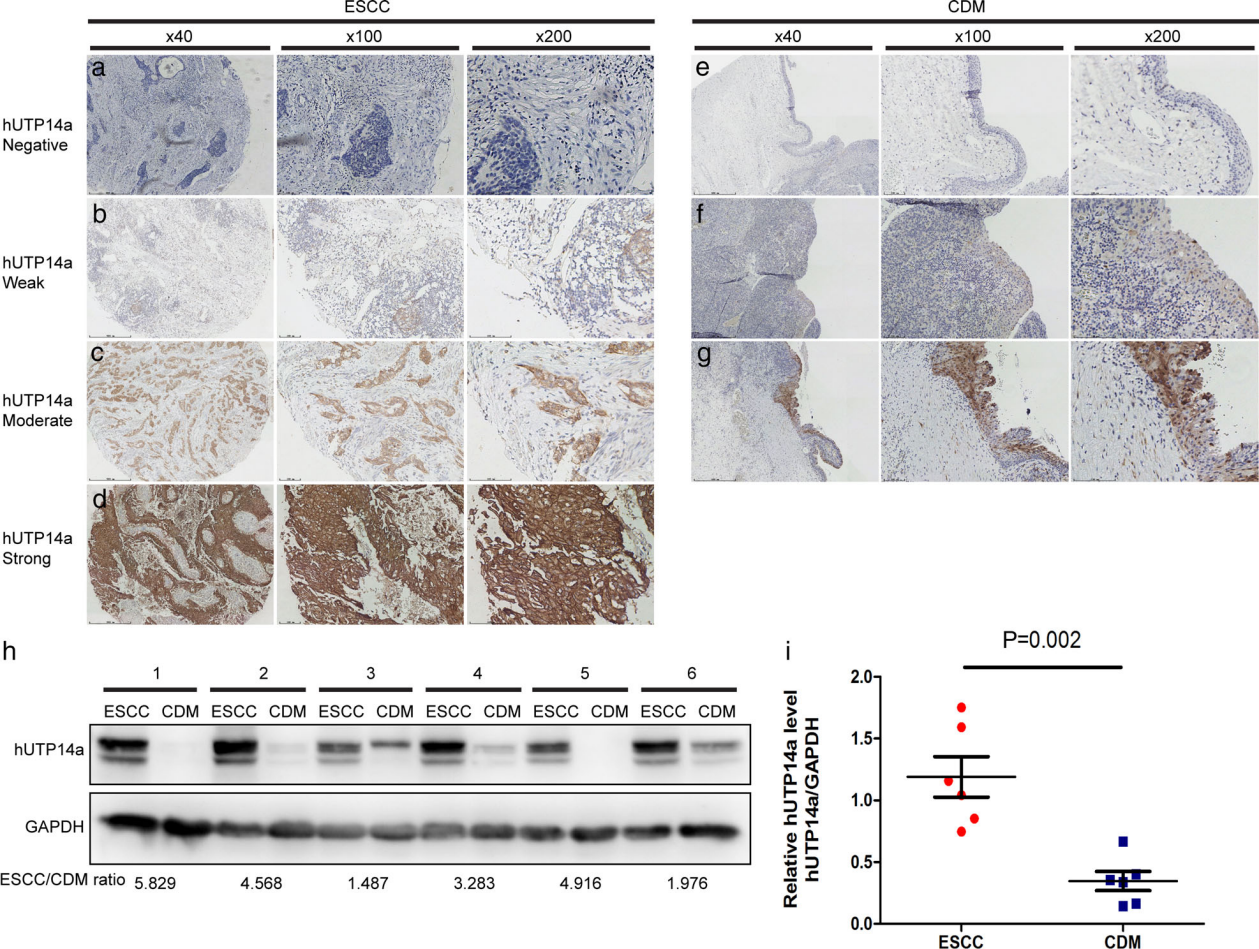
IHC staining and Western blot analysis of UTP14a in esophageal squamous cell carcinoma (ESCC) and CDM tissues. (**a–d**) Negative to strong UTP14a expression in ESCC tissues. (**e–f**) Negative‐to‐moderate UTP14a expression in CDM tissues. (**h**) Western blot analysis of six pairs of ESCC and CDM tissue. (**i**) Relative UTP14a protein levels were quantified using Quantity One software.

As shown in Fig 1a–g, in ESCC tissues, UTP14a was mainly located in the cytoplasm of tumor cells, and only a small part was located in the nuclei. UTP14a was positively expressed in 88.1% (185/210) of the ESCC samples. In CDM tissues, UTP14a was also mainly localized in the cytoplasm of squamous epithelial cells, with positive staining occurring in nine cases (9/30, 30%). The expression of UTP14a in ESCC was significantly higher than in CDM samples (*P* < 0.001, Table 1). These results were further confirmed by Western blot analysis (*P* = 0.002; Fig 1h,i).

**Table 1:** Correlation between UTP14a immunostaining and the clinical characteristics in 210 ESCC patients

		UTP14a expression				
Factor	Total	Negative	Weak	Moderate	Strong	*P*
Gender						0.621*
Male	164	19	38	49	58	
Female	46	6	9	18	13	
Age (years)	61.2 ± 8.1	59.6 ± 9.9	62.8 ± 7.3	61.6 ± 7.9	60.5 ± 8.1	0.313#
Smoking history						0.624*
No	68	10	13	24	21	
Yes	142	15	34	43	50	
BMI (kg/m^2^)	21.7 ± 3.0	21.4 ± 2.9	21.3 ± 2.9	22.0 ± 3.5	21.7 ± 2.6	0.664#
Tumor location						
Upper thoracic	42	3	11	12	8	0.080*
Middle thoracic	139	13	30	44	52	
Lower thoracic	29	9	6	8	11	
Grade						
Well differentiated (G1)	59	7	16	23	13	0.380*
Moderately differentiated (G2)	113	12	21	35	45	
Poorly differentiated (G3)	38	6	10	9	13	
pT						0.005*
Tis	4	3	0	1	0	
T1	5	1	1	3	0	
T2	29	8	7	4	10	
T3	143	11	32	50	50	
T4	29	2	7	9	11	
pN						0.307*
N0	102	16	24	31	31	
N1	66	7	15	19	25	
N2	34	1	7	16	10	
N3	8	1	1	1	5	
Distant metastasis (M)						>0.999*
M0	210	25	47	67	71	
M1	0	0	0	0	0	
pStage						0.001*
0	4	3	0	10	0	
I	19	6	5	3	5	
II	82	9	15	30	28	
III	105	7	27	33	38	
Tissue origin						<0.001*
ESCC	210	25	47	67	71	
CDM	30	21	5	4	0	

T, tumor stage (depth of invasion); N, lymphatic dissemination stage, based on the eighth Edition of the American Joint Committee on Cancer (AJCC) staging protocol.^14^ pT, primary tumour; Tis, tumour in situ; N0, no positive lymph nodes; N1, 1–2 positive lymph nodes; N2, 3–6 positive lymph nodes; N3, > 6 positive lymph nodes; ESCC, esophageal squamous cell carcinoma; CDM, cancer distant mucosa.

* *x* ^2^ test;

# ANOVA test.

### Relationship between UTP14a expression and clinical characteristics

Among the 210 patients, 164 were male, and the male:female ratio was 3.6:1 (Table 1). Gender, age, smoking history, and body mass index (BMI) were well balanced. High expression of UTP14a in ESCC was significantly correlated with postoperative tumor invasive depth (pT stage) (*P* = 0.005) and advanced TNM stage (*P* = 0.001). However, no significant correlation was found between UTP14a expression and clinical characteristics such as tumor location, differentiation grades, and postoperative lymphatic dissemination stage (pN stage).

### UTP14a expression and survival rates

**Figure 2:**
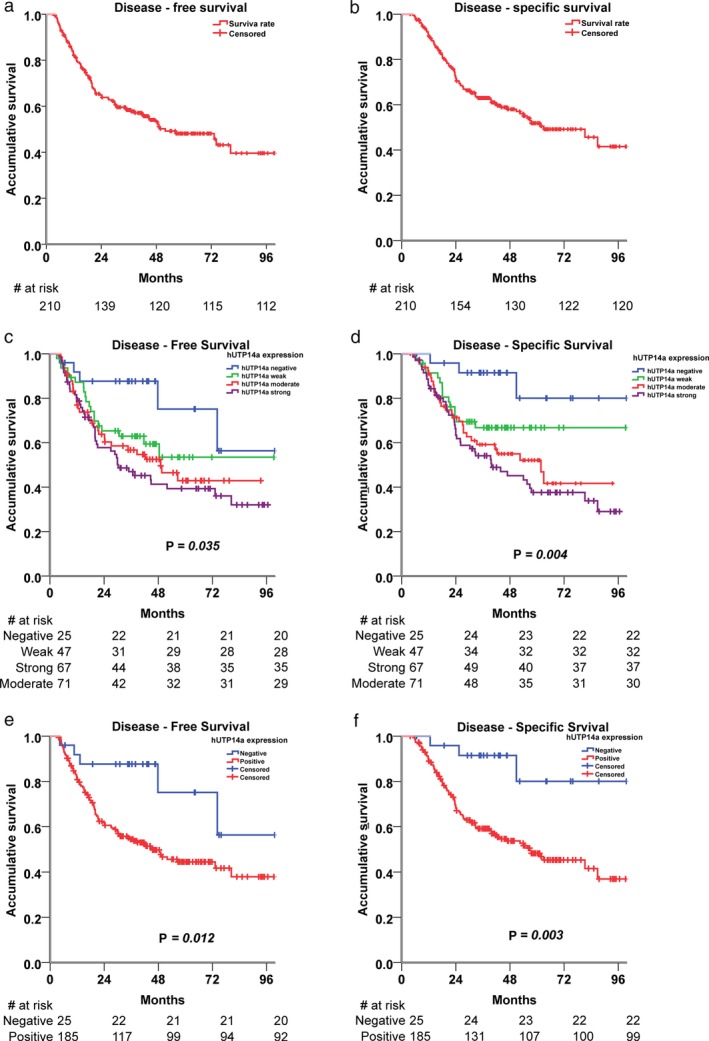
Kaplan–Meier survival curves for (**a**) DFS and (**b**) DSS of 210 patients. (**c**) DFS and (**d**) DSS of different UTP14a expression classifications. (**e,f**) Compared with UTP14a negative group, the UTP14a positive group was significantly higher in DFS and DSS (*P* = 0.012 and *P* = 0.003, respectively). Data were compared using the log‐rank test.

The DFS rate was 82.1% at one year, 58.4% at three years, 48.1% at five years, and 39.6% at eight years, whereas the DSS rate was 90.8% at one year, 63.0% at three years, 51.9% at five years, and 41.5% at eight years (Fig 2a,b). As shown in Fig 2c,d, the DFS and DSS were significantly different among the negative and strong expression of UTP14a (*P* = 0.035 and *P* = 0.004, respectively). In Fig 2e,f, compared with UTP14a negative group, UTP14a positive group was significantly higher in DFS and DSS (*P* = 0.012 and *P* = 0.003, respectively), and high UTP14a expression may indicate poor prognosis.

Prognostic factors for DFS and DSS are presented in Table 2. Univariate analysis indicated that age, gender, smoking history, BMI, and tumor location did not significantly influence DFS and DSS; only the differentiation grade, pT stage, pN stage, and UTP14a expression were associated with the DFS and DSS rates. Moreover, similar findings were observed after multivariate Cox proportional hazards regression analysis, which demonstrated that the differentiation grade, pT stage, pN stage, and UTP14a expression were significantly correlated with patients' DFS and DSS; higher expression of UTP14a predicted poorer DFS and DSS.

**Table 2:** Risk factors for DFS and DSS by univariate and multivariate Cox proportional hazards regression analyses

	For DFS		For DSS	
Variable	Hazard ratio (95% CI for HR)	*P*	Hazard ratio (95% CI for HR)	*P*
Univariate				
Gender	0.990 (0.615–1.593)	0.966	0.918 (0.557–1.513)	0.738
Age	1.010 (0.984–1.037)	0.453	1.002 (0.975–1.029)	0.904
Smoking history	0.741 (0.491–1.119)	0.154	0.807 (0.525–1.240)	0.328
BMI	1.001 (0.932–1.074)	0.980	0.985 (0.915–1.061)	0.697
Tumor location	0.715 (0.501–1.020)	0.064	0.689 (0.474–1.003)	0.052
Differential grade	1.639 (1.199–2.241)	0.002	1.525 (1.106–2.103)	0.010
pT stage	1.535 (1.245–1.892)	0.000	1.514 (1.216–1.884)	0.000
pN stage	1.823 (1.473–2.256)	0.000	1.901 (1.528–2.365)	0.000
pTNM stage	1.593 (1.367–1.855)	0.000	1.645 (1.398–1.936)	0.000
UTP14a expression	1.351 (1.096–1.664)	0.005	1.503 (1.197–1.886)	0.000
Multivariate*				
Differential grade	1.585 (1.139–2.205)	0.006	1.548 (1.110–2.160)	0.010
pT stage	1.436 (1.140–1.809)	0.002	1.400 (1.097–1.787)	0.007
pN stage	1.713 (1.368–2.145)	0.000	1.794 (1.423–2.262)	0.000
UTP14a expression	1.316 (1.059–1.635)	0.013	1.461 (1.154–1.849)	0.002

* Cox proportional hazards regression analysis by step forward. UTP14a expression, U Three Protein 14a expression.

### Inhibition of UTP14a inhibited cell proliferation and invasion in ECA109 cells

**Figure 3:**
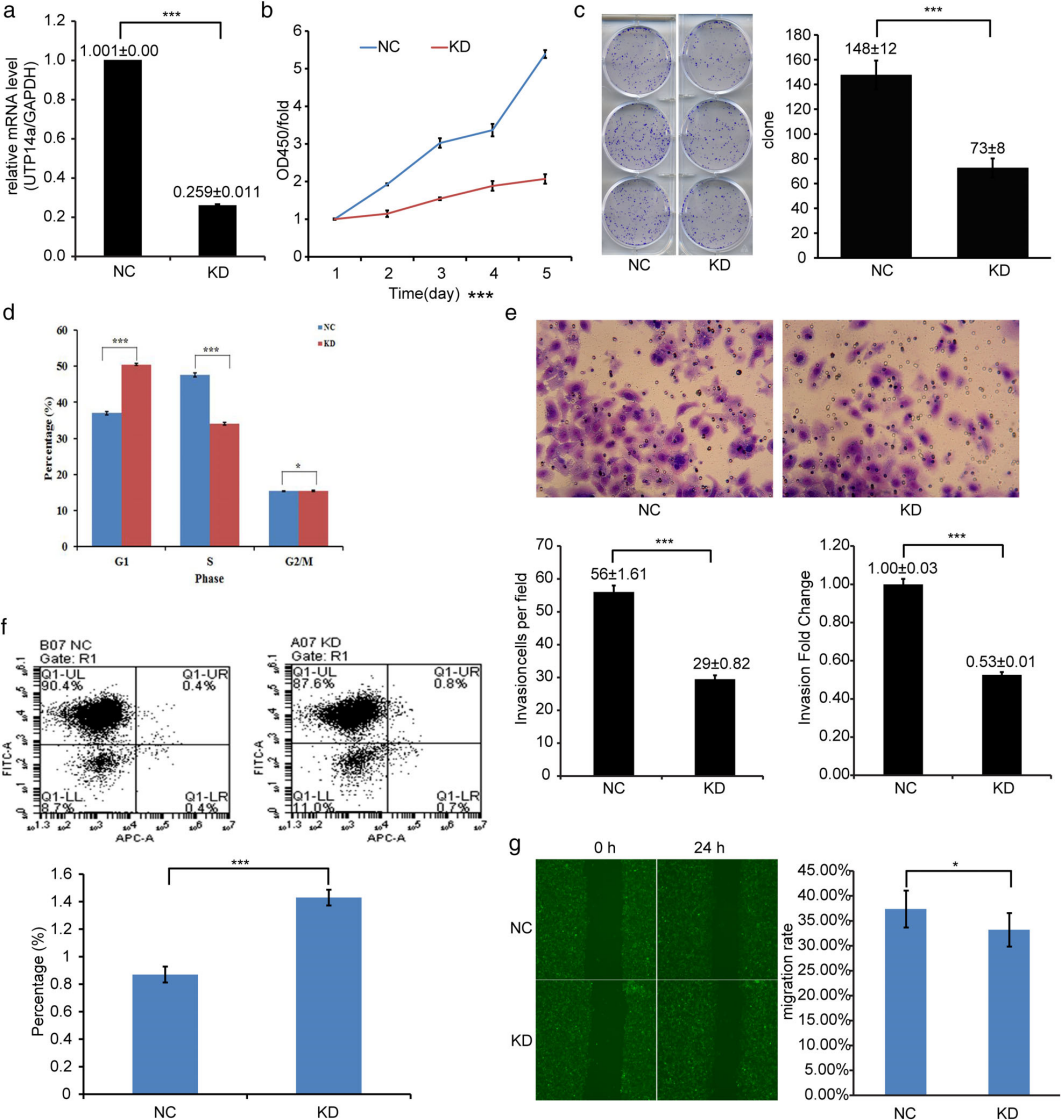
UTP14a knockdown experiments in ECA109 cells. (**a**) ECA109 cells were infected with UTP14a‐siRNA lentivirus (KD) and negative control lentivirus (NC). qRT‐PCR results show that the interference efficiency was 74.1% (***P < 0.001). (**b**) Results of the CCK8 assay. The cell proliferation of KD cells was significantly slower than that of NC cells (day 5, ***P < 0.001). (**c**) Results of the cell colony formation assay. The cell count in the KD group was significantly lower than in the NC group (***P < 0.001). (**d**) Results of the PI‐FACS cell cycle analysis. After five days of cell culture, the proportion of KD cells in the G0/G1 phase was significantly larger, and that in the S phase was significantly smaller than the respective proportions of NC cells (*P > 0.05, ***P < 0.001,). (**e**) Results of the cell invasion assay. After 72 hours, the number of invasive cells in the KD group was significantly lower than that in the NC group (***P < 0.001). (**f**) Results of the cell apoptosis assay. After five days of cell culture, the percentage of apoptotic cells in the KD group was higher than in the NC group(***P < 0.001), yet both percentages were below five, so no apoptosis occurred in the two groups. (**g**) Results of the Celigo wound healing assay. After 24 hours, no significant difference was found between the two groups (*P > 0.05).

The results of the UTP14a knockdown experiments in ECA109 cells are shown in Fig 3. The interference efficiency of UTP14a‐siRNA lentivirus is 74.1% (Fig 3a). The cell proliferation of KD‐transfected cells was significantly slower than that of NC‐transfected cells according to the CCK8 and cell colony formation assays (Fig 3b,c). PI‐FACS cell cycle analysis showed that, after 5 days of cell culture, the proportion of KD cells in the G0/G1 phase was significantly larger, and that in the S phase was significantly smaller, than the respective proportions of NC cells (Fig 3d).

As shown in Fig 3e, after 72 hours, the number of invasive cells in the KD group was significantly lower than that in the NC group (*P* < 0.001). The results of the cell apoptosis assay, after five days of cell culture, are shown in Fig 3f. Although the percentage of apoptotic KD cells was higher than that of NC cells, all percentages were below five, so no apoptosis occurred in the two groups. The results of the Celigo wound healing assay are shown in Fig 3g. No significant difference was found between the two groups (*P* = 0.098).

## Discussion

WIG‐1, which encodes an RNA zinc finger protein, is known to be a p53 target gene. Our previous research found that WIG‐1 could effectively reverse the malignant phenotype, suppress cell proliferation, and induce G0/G1 cell cycle arrest and apoptosis of EC109 cells under zinc deficiency conditions.^5^, ^6^ These results were confirmed by Bersani *et al*.^18^ who suggested that WIG‐1 binds to the FAS mRNA 3'‐UTR and decreases its stability through AU‐rich elements. Knockdown of WIG‐1 can increase cell death and reduce cell cycle arrest upon DNA damage. UTP14a was found to interact directly with WIG‐1 in a yeast‐two‐hybrid system and is thought to be a downstream target of WIG‐1 (K.K. Li *et al*., unpublished data). UTP is encoded by U3 snoRNA, which is found predominantly in the nucleolus.^19^ UTP14 is a subunit of the U3 containing small subunit (SSU) processome complex, and interacts with the preribosome. As a member of the UTP14 family, UTP14a is also involved in ribosome biogenesis and 18S rRNA synthesis.^7^, ^8^ Some studies found that the expression of UTP14a is upregulated in human hepatocellular carcinoma and colorectal carcinoma, and it may be associated with poor prognosis.^12^, ^13^

In this study, UTP14a was positively expressed in 88.1% of the ESCC samples, and 30% of CDM samples exhibited positive expression; Western blot analysis further confirmed that the protein levels of UTP14a were significantly higher in ESCC. Nucleolar proteins participate in ribosome biogenesis; some studies further demonstrated that nucleolar proteins may interact with many tumor suppressors and proto‐oncogenes, and may be associated with many types of cancer.^20^, ^21^ In ESCC tissues, UTP14a was mainly located in the cytoplasm of tumor cells and only lowly expressed in the nuclei. The potential roles of UTP14a in ESCC remain unclear.

In our previous research, we found that UTP14a is a downstream molecular target of WIG‐1 and may play an important role in the p53 pathway. This was confirmed by Hu *et al*., who found that UTP14a interacts with p53; overexpression of UTP14a could lead to p53 protein degradation, and knockdown of UTP14a could cause cell cycle arrest and apoptosis in HeLa cells.^9^ Zhang *et al*.^13^ found that UTP14a is upregulated in human colorectal cancer tissues. They demonstrated that UTP14a forms a complex with USP36/Fbw7γ, stabilizes c‐Myc in the nucleolus, and inhibits c‐Myc degradation; knockdown of UTP14a leads to a decrease in c‐Myc levels and inhibits tumor growth. In colorectal cancer cell lines, Liu *et al*. demonstrated that UTP14a acts as a ubiquitin E3 ligase for tumor suppressor retinoblastoma (RB); overexpression of UTP14a leads to protein degradation of both p53 and RB, and causes cancer cell proliferation.^22^

All 210 patients in this study were operated on by the same surgeon using a uniform surgical approach so that the results were more reliable. The five‐year DFS and DSS of these patients were 48.1% and 51.9%, respectively, and the eight‐year DFS and DSS were 39.6% and 41.5%, respectively. High expression of UTP14a in ESCC was significantly correlated with the pT stage and pTNM stage; overexpression of UTP14a indicated an increased risk of tumor proliferation and invasion. Meanwhile, from the Kaplan–Meier survival curves and the log‐rank test, we found that high UTP14a expression predicts poor DFS and DSS. Data from the Worldwide Esophageal Cancer Collaboration database show that the pTNM stage is strongly associated with survival,^23^ and the results of our univariate and multivariate Cox proportional hazards regression analyses further confirmed that UTP14a levels and the pTNM stage are independent predictors of long‐term survival. Furthermore, our in vitro experiments showed that upon knockdown of UTP14a, cell proliferation and cell invasion were decreased significantly. Therefore, UTP14a expression levels might be indicative of the aggressiveness of ESCC, and might be useful as a prognostic marker to predict the occurrence of ESCC and death. We speculate that UTP14a might work as a tumor promoter through the cell proliferation and cell invasion pathways, but this hypothesis requires confirmation by further studies.

Although our samples were retrospectively collected from a single institution, the database in our department and the patient follow‐up system were well established, and the surgical procedures, pathologic examinations, and patient follow‐up were highly uniform throughout the entire study period. Additionally, all patients enrolled in this study had ESCC and the operations were performed by a single surgeon using a uniform surgical approach, with standard exclusion and inclusion criteria. Therefore, we believe that our results are robust and valid.

In conclusion, our results suggest that UTP14a expression is correlated with tumor proliferation and invasion; higher expression of UTP14a is a predictor of poor prognosis in patients with ESCC. Further studies with larger cohorts are needed to validate our results, and it will be interesting and useful to investigate the underlying mechanisms.

## Disclosure

There were no potential conflicts of interest, including specific financial interests and relationships in our manuscript, and the authors have no conflicts of interest.
